# Clinical and laboratory features of urinary tract infections in young infants

**DOI:** 10.1590/1678-4685-JBN-3602

**Published:** 2018-04-26

**Authors:** Denise Swei Lo, Larissa Rodrigues, Vera Hermina Kalika Koch, Alfredo Elias Gilio

**Affiliations:** 1 Universidade de São Paulo, Hospital Universitário, Departamento de Pediatria, São Paulo, SP, Brasil.; 2 Universidade de São Paulo, Faculdade de Medicina, Instituto da Criança, Departamento de Pediatria, São Paulo, SP, Brasil.

**Keywords:** urinary tract infections, urinalysis, Escherichia coli, C-Reactive protein, pyuria, leukocyte count, infecções urinárias, urinálise, Escherichia coli, proteína C reativa, piúria, contagem de leucócitos

## Abstract

**Introduction:**

Urinary tract infection (UTI) is the most common serious bacterial infection in young infants. Signs and symptoms are often nonspecific.

**Objectives:**

To describe clinical, demographic and laboratory features of UTI in infants ≤ 3 months old.

**Methods:**

Cross-sectional study of infants ≤ 3 months old with UTI diagnosed in a pediatric emergency department, for the period 2010-2012. UTI was defined as ≥ 50,000 colony-forming units per milliliter of a single uropathogen isolated from bladder catheterization. Paired urinalysis and urine culture from group culture-positive and group culture-negative were used to determine the sensitivity and specificity of pyuria and nitrite tests in detecting UTI.

**Results:**

Of 519 urine cultures collected, UTI was diagnosed in 65 cases (prevalence: 12.5%); with male predominance (77%). The most common etiologies were *Escherichia coli* (56.9%), *Klebsiella pneumoniae* (18.5%) and *Enterococcus faecalis* (7.7%). Frequent clinical manifestations were fever (77.8%), irritability (41.4%) and vomiting (25.4%). The median temperature was 38.7°C. The sensitivity of the nitrite test was 30.8% (95%CI:19.9-43.4%), specificity of 100% (95%CI:99.2-100%). Pyuria ≥ 10,000/mL had a sensitivity of 87.7% (95%CI:77.2-94.5%), specificity of 74.9% (95%CI:70.6 -78.8%). The median peripheral white blood cell count was 13,150/mm^3^; C-reactive protein levels were normal in 30.5% of cases.

**Conclusions:**

The male: female ratio for urinary tract infection was 3.3:1. Non-*Escherichia coli* etiologies should be considered in empirical treatment. Fever was the main symptom. Positive nitrite is highly suggestive of UTI but has low sensitivity; whereas pyuria ≥ 10,000/mL revealed good sensitivity, but low specificity. Peripheral white blood cell count and C-reactive protein concentration have limited usefulness to suggest UTI.

## RESUMO

**Introdução:**

A infecção do trato urinário (ITU) é um quadro infeccioso grave mais frequente em lactentes jovens, cujos sinais e sintomas são frequentemente inespecíficos.

**Objetivos:**

Descrever aspectos clínicos, demográficos e laboratoriais de ITU em lactentes ≤ 3 meses.

**Métodos:**

Estudo transversal de ITU diagnosticada em lactentes ≤ 3 meses, em pronto-socorro geral de pediatria, entre 01/01/2010 a 31/12/2012. Diagnóstico de ITU definida como crescimento ≥ 50.000 unidades formadoras de colônia por mililitro de uropatógeno único, colhido por cateterismo vesical. Urina tipo I e urocultura foram pareadas dos grupos cultura-positiva e cultura-negativa para determinar a sensibilidade e especificidade de piúria e teste do nitrito para o diagnóstico de ITU.

**Resultados:**

Das 519 uroculturas colhidas, confirmou-se 65 casos de ITU (prevalência: 12,5%), com predomínio em meninos (77%). As etiologias mais frequentes foram *Escherichia coli* (56,9%), *Klebsiella pneumoniae* (18,5%) e *Enterococcus faecalis* (7,7%). Os sintomas mais frequentes foram febre (77,8%), irritabilidade (41,4%) e vômitos (25,4%). A temperatura mediana foi de 38,7°C. A sensibilidade do nitrito positivo foi de 30,8% (IC95%:19,9-43,4%), especificidade de 100% (IC95%:99,2-100%). Piúria ≥ 10.000/mL apresentou sensibilidade de 87,7% (IC95%:77,2-94,5%), especificidade de 74,9% (IC95%:70,6-78,8%). A contagem mediana de leucócitos foi 13.150/mm^3^. A proteína C reativa foi normal em 30,5% dos casos.

**Conclusões:**

A proporção entre meninos e meninas para ITU foi de 3,3:1. Outros agentes além de *Escherichia coli* devem ser considerados no tratamento empírico. A febre foi a principal queixa. O nitrito positivo é altamente sugestivo de ITU mas tem baixa sensibilidade; enquanto que a piúria ≥ 10.000/mL revelou boa sensibilidade, porém baixa especificidade. Leucograma e proteína C reativa demonstraram pouca utilidade clínica para sugerir ITU.

## INTRODUCTION

Urinary tract infection (UTI) has been described as the most frequent severe bacterial infection in infants below the age of three months.^1^^-^ 4 A meta-analysis by Shaikh N et al.^5^ showed that the prevalence of UTI in young infants with fever was 7.5% in girls and ranged from 2.4% in circumcised boys to 20.1% in non-circumcised boys. The major challenge is that signs and symptoms at this age tend to be nonspecific.^3^^,^ 6 As has been reported, fever is the main symptom, followed by irritability, lethargy, vomiting, diarrhea, anorexia, jaundice and low weight gain.^3^^,^ 6 ^,^ 7

Young infants are at a higher risk for developing pyelonephritis, especially if the diagnosis and adequate antimicrobial therapy are delayed.^6^^,^ 8 ^-^ 11 There is an association between delayed therapy of febrile UTI and an increased risk of progression resulting in renal scarring.^11^^,^ 12 Such cases tend to progress to hypertension and renal failure.^13^ Young infants, whose immune systems is still maturing, are particularly vulnerable to bacteremia associated with pyelonephritis.^14^ The definitive diagnosis of UTI is based on finding significant bacteriuria in a quantitative urine culture, which may takes days to yield a final result. Thus, empirical therapy should be initiated if UTI is suspected in clinical data and laboratory tests such as urinalysis and/or bacterioscopy of urine. Other tests often carried out to assess the risk of bacterial infection in febrile infants, such as the white blood cell count and the reactive C-reactive protein (CRP) test are of questionable value for a presumptive diagnosis of pyelonephritis. Studies in young infants, however, are lacking.^15^^,^ 16 Furthermore, the American Academy of Pediatrics has excluded infants aged below 2 months in its recommendations for the therapy of UTI.^17^^,^ 18

This epidemiological review aims to describe the clinical and laboratory findings of UTI in infants aged below 3 months in an urban community. The diagnosis of this condition is enhanced by understanding the prevalence of UTI and the main clinical and laboratory findings in urine and blood tests at this age group. A description of the main causative agents and the antimicrobial susceptibility profile may help chose the best therapy in our context.

## METHOD

### TYPE OF STUDY AND CONTEXT

An epidemiological cross-sectional cohort study was conducted at the University Hospital of University of Sao Paulo, a secondary level hospital that serves a population of about 500 thousand people in the Western region of São Paulo. The study site was the Pediatrics Emergency Unit. The study was undertaken from 1^st^ January 2010 to 31^st^ December 2012; during this period, 188,440 cases of children aged 0-15 years were seen.

### LABORATORY METHODS AND DEFINITION OF UTI

Urine samples were collected by urinary bladder catheterization. Antisepsis was carried out with 1% aqueous. Urine samples were immediately sent to the hospital laboratory; urine cultures were plated onto blood agar medium and MacConkey agar (Plastlabor^®,^ Rio de Janeiro). The Vitek (Bio-Mérieux^®^) was used to identify and test the susceptibility of strains. UTI was established when a quantitative urine culture of urine collected by urinary bladder catheterization yielded growth of ≥ 50,000 colony forming units (CFU)/mL of a single microbial agent.

Urine nitrite was tested in urinalysis samples for positivity using reagent strips (Multistix^®^ 10 SG - Siemens) in semiautomatic equipment (Clinitek Advantus - Siemens), based on the light reflectance spectrophotometry. Urine sediment microscopy of centrifuged urine was done with a common optical microscope at 10x and 40x magnification; white blood cells were observed and quantified by using a Neubauer chamber.

### STUDY POPULATION AND METHOD

All infants aged from 0 to 3 months that sought the emergency unit and underwent urine culture tests were enrolled, on medical criteria. Demography data (sex and age), clinical and laboratory findings of infants with confirmed UTI were investigated. Signs and symptoms in order of frequency were: fever, maximum verified temperature, irritability, vomiting, inadequate food intake, dehydration, low weight gain, jaundice, constipation, diarrhea and altered urine. These were itemized as percentages over the total number of recordings found in patient registries. Laboratory data consisted of: urinalysis (quantifying urinary white blood cells and a positive nitrite test), a red blood count and a C-reactive protein (CRP). The white blood cell count and CRP were described as the median, minimal and maximal values. Microorganisms found in quantitative urine cultures were expressed as number of episodes and percentages. All episodes of UTI caused by urine micropathogens were added and assessed regarding susceptibility to commonly used antimicrobial agents.

Urinalysis test results in young infants without UTI during the study period were used for comparison purposes with the positive UTI group. The chi-square test and Fisher's exact test were used for comparing the groups. The sensitivity, specificity, positive predictive value (PPV) and the negative predictive value (NPV) of a cutoff point for white blood cells (WBC) of ≥ 10,000 WBC/mL in urinalysis and a positive nitrite test were evaluated as criteria for diagnosing UTI. These parameters were expressed in 95% confidence intervals (CI 95%). Exclusion criteria were: incomplete identification or laboratory data in patient registries and test sampling of the same patient twice on the same day.

### ETHICS

The research ethics committee of the Hospital approved this epidemiological study (registry number 622/05).

## RESULTS

**Figure 1:**
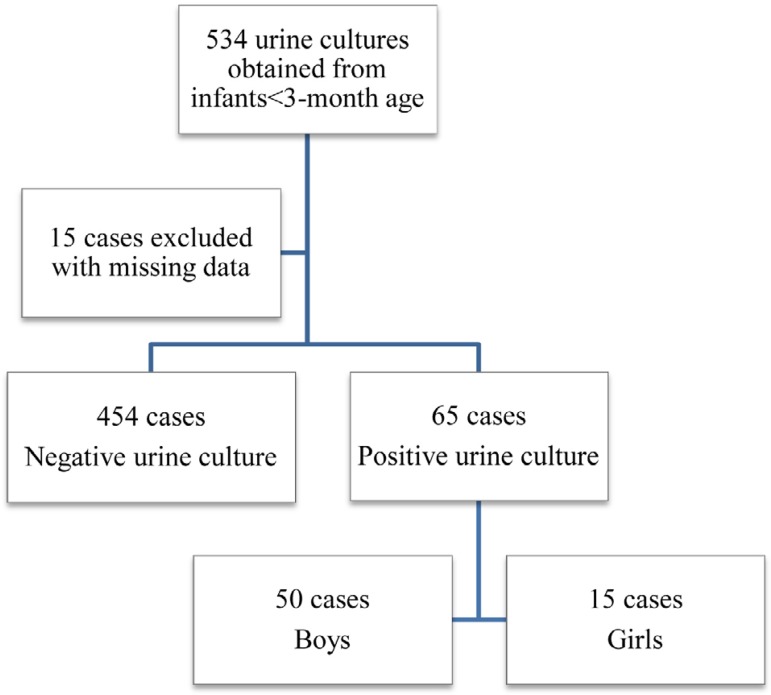
Flowchart of the cohort.

Figure 1 presents the series during the 3-year study period. A total of 534 urine cultures of 501 infants aged less than 3 years with suspected UTI were done. In this group, one urine culture was carried out in each of 470 infants, two urine cultures were done in each of 29 infants, and three urines cultures were done in each of 2 infants. Incomplete patient identification and/or laboratory data led to 15 patients being excluded from the study. OF 519 episodes that were investigated, 454 urine cultures were negative, and 65 urine cultures were positive. Thus, the full number of cases of UTI in our statistical analysis was 65, in 64 infants. One male patient had two UTI episodes caused by *E. coli* with a 19-day interval between each episode.

The prevalence of UTI was 12.5%. The male to female ratio was 3.3: 1. Table 1 presents the clinical and laboratory findings of infants with UTI, aged below 3 years. The median temperature of fever was 38.7°C; 64.9% subjects had temperatures below 39°C. Supplementary tests were the complete blood count (done in 60 cases), in which the median value of WBC was within normal limits (13,150/mm^3^). The C-reactive protein (CRP) test was done in 36 cases and was within normal limits in 30.5% of samples. Urinalysis was done in all 65 cases. Comparing the positive UTI and negative UTI groups with the chi-square test showed that a cutoff point of white blood cells (WBC) in urine of more than 10,000 WBC/mL had a sensitivity of 87.7% (CI 95%: 77.2%-94.5%), a specificity of 76.6% (CI 95%: 72.5%-80.5%), a positive predictive value (PPV) of 35.0% (CI 95%: 27.7%-42.8%), and a negative predictive value (NPV) of 97.7% (CI 95%: 95.6%-99.0%). This comparison applied to a positive nitrite test showed a sensitivity of 30.8% (CI 95%: 19.9%-43.4%), a specificity of 100% (CI 95%: 99.2%-100%), a PPV of 100% (CI 95%: 83.2%-100%), and a NPV of 91.0% (CI 95%: 88.1%-93.3%). There were no cases of bacteremia due to UTI in the 63 cases in whom blood cultures were carried out.

**Table 1:** Clinical and laboratory findings of infants aged below 3 months with urinary tract infection

Variable	
Male sex	50 (76.9%)
Age in months ± SD, median	1.5 ± 0.7
Maximum fever temperature	39.5°C
Median temperature ± SD	38.7°C ± 0.5°C
White blood cells/mm^3^ ± SD	13150 ± 5852
Blood counts with white blood cells ≥ 15,000/mm^3^	33.30%
CRP (mg/L), median	17.5
CRP < 5mg/L	30.5%
Urinary white blood cells > 10,000/mL	57 (87.7%)
Positive nitrite	20 (30.8%)

SD: standard deviation; CRP: C-reactive protein

Signs and symptoms are shown in Table 2. Percentages were calculated over the full number of registries of each datum. Information retrieval was possible in 89.2 to 96.9% of patient registries. The main symptom was fever without locating signs, which was found in 77.8% of cases; it was followed by irritability (41.4%) and vomiting (25.4%). Parents or caretakers reported changes in urine in only 10.3% of cases. No cases presented with jaundice.

**Table 2:** Sign and symptoms of infants aged below 3 months with urinary tract infection

Signs and symptoms	No. of cases/No. registries	%
Fever without localizing signs	49/63	77.8
Irritability	24/58	41.4
Vomiting	15/59	25.4
Low food intake	12/58	20.7
Clinical dehydration	11/58	19.0
Low weight gain	8/58	13.8
Constipation	7/58	12.1
Diarrhea	4/58	6.9
Urinary changes	6/58	10.3

Table 3 presents the causative microbial agents that were found in our cohort. The most frequent agent was *E. coli*, found in 56.9% of cases, followed by *Klebsiella pneumoniae* (18.5%) and *Enterococcus faecalis* (7.7%). Of 5 UTI cases due to *E. faecalis* (infants aged 8, 11, 19, 27 and 50 days), 4 were newborn. The sensitivity of these microbial agents was higher than or equal to 80% to the following antimicrobials: aminoglycosides (gentamycin: 80%, amikacin: 86.2%), third-generation cephalosporins (cefotaxime: 84.6%, ceftazidime: 89.2%), fourth-generation cephalosporins (cefepime: 87.7%). *E. faecalis* were all sensitive to ampicillin. A few urinary pathogens had low sensitivity to commonly used antimicrobial agents in the empirical treatment of UTI in older children, such as: first-generation cephalosporin (cephalothin: 63.1%), sulfamethoxazole-trimethoprim (60%), and amoxicillin-clavulanate (73.8%).

**Table 3:** Etiological agents in infants aged below 3 months with urinary tract infection

Etiological agents	n	%
*Escherichia coli*	37	56.9
*Klebsiella pneumoniae*	12	18.5
*Enterococcus faecalis*	5	7.7
*Enterobacter aerogenes*	3	4.7
*Proteus mirabilis*	2	3.1
*Enterobacter cloacae*	2	3.1
*Staphylococcus epidermidis*	1	1.5
*Raoultella planticola*	1	1.5
*Serratia marcescens*	1	1.5
*Pantoea spp*	1	1.5
Total	65	100

## DISCUSSION

This study aims to bring clinical and laboratory knowledge about UTI in infants aged below 3 months in an urban community. Among this cohort, we found a high prevalence of UTI (12.5%) over the total number of urine cultures done in this group. There were three times more cases among females, concurring with previous studies.^1^^,^ 6 ^,^ 9 ^,^ 10 ^,^ 19 ^,^ 20 Fever without localizing signs was the main presenting sign (77.8% of cases). As in previous papers,^15^^,^ 21 the complete blood count and the CRP were of little clinical use in the diagnosis of UTI; the white blood counts were within normal limits, and 30.5% of CRP tests were within normal. The median CRP test value was lower than 20 mg/L, a number that has been used by some authors to suggest a diagnosis of pyelonephritis.^15^^,^ 21 In the urinalysis, a positive nitrite test was highly specific and had a high PPV; therefore, if initial screening reveals a positive nitrite test, antimicrobial agents should be started immediately after collecting samples for quantitative urine cultures. The nitrite test, however, had low sensitivity and should not be used to discard UTI. On the other hand, a white blood cell (WBC) cutoff value of ≥ 10,000/mL (equal to 10 WBC/mm^3^) was reasonably sensitive for a presumptive diagnosis of UTI; its specificity and PPV, however, were low.^21^^,^ 22 In our cohort, starting empirical therapy using a white blood cell count of ≥ 10,000/mL as a cutoff point would results in unnecessary treatment of 65.0% of cases.

UTI should not be suspected base on urinary complaints, which were present in only 10.3% of our cohort, or on high fever, which was often below 39°C in 64.9% of our cohort. Our findings agree with previous papers that report fever as the main symptom in young infants with non-specific clinical findings.^1^^,^ 3 ^,^ 6 ^,^ 7 No patient presented with jaundice in our cohort; it has been reported previously.^7^^,^ 23 In this age group, some authors have defined UTI as present when there are more than 50,000 CFU/mL of a single pathogen, or ≥ 10,000 CFU/mL together with changes in urinalysis.^3^^,^ 24 The American Academy of Pediatrics included an association between a qualitative urine culture with over 50,000 CFU/mL and urinary bacteria and/or white blood cells in urine as a criterion for diagnosing UTI in the 2 to 24 month age group.^18^^,^ 19 In our cohort, 8 cases (12.3%) had urinary white blood cell counts below 10,000/mL and a negative nitrite test, but with quantitative urine culture showing over 50,000 CFU/mL of a single type of bacteria. In all of these cases, urine samples were collected based on clinical findings (fever, irritability, vomiting, anorexia, low weight gain, dehydration or urinary changes); these cases should not be classified as asymptomatic bacteriuria. Therefore, it is important to carry out urinalysis and urine cultures sampled by bladder catheterization in young infants. This recommendation concurs with the findings in Shaikh *et al.'s* 25 paper, which reported that about 10% of children had urinary symptoms and confirmed UTI by urine cultures even though the white blood cell count in urinalysis was within normal limits. This is seen more frequently in UTI cases caused by *Enterococcus*, *Klebsiella* and *P. aeruginosa* species, all of which are relevant in young infants. On the other hand, if the urinary white blood cell count was ≥ 10,000/mL in urinalysis, a negative urine culture was important to eventually interrupt antimicrobial therapy.

*E. coli* was the main microorganism in our cohort - 56.9% of cases. This prevalence was lower than previously published results in this age group (62%-88%),^1^^,^ 4 ^,^ 6 ^,^ 9 ^,^ 10 ^,^ 17 but higher than the 41.7% prevalence reported by Chen *et al.* 23 in young infants with elevated blood bilirubin secondary to UTI. Other microorganisms that should be considered when initiating empirical antimicrobial therapy are: *K. pneumoniae* (18.5%) and *E. faecalis* (7.7%). The choice of empirical antimicrobial therapy should take into account updated information about the prevalence of urinary pathogens for each age, sex and place.^17^ According to the sensitivity profiles of such urinary pathogens, it is appropriate to start empirical therapy with an aminoglycoside (amikacin) or a third-generation cephalosporin, associated with ampicillin if there are Gram-positive cocci (*E. faecalis*), especially in neonates.

The purpose of this study was to assess the presenting clinical and laboratory findings of young infants, not including the progression of therapy and laboratory testing. It is relevant to consider that UTI may be the first sign that a child has a congenital anomaly of the kidney or urinary tract.^6^^,^ 8 ^,^ 10 Given the retrospective nature of this study, we were unable to accurately characterize imaging methods in the evaluation of anatomical changes of the urinary tract in our cohort. We also had no access to prenatal ultrasounds of these infants to check for any diagnosis of renal and urinary malformations. Urine bacterioscopy, which could have been useful in the initial approach of these cases while waiting for quantitative urine cultures, was not carried out in our study.^18^^,^ 19 We were unable to accurately gather specific types of clinical data, such as recent use of antimicrobial medication, which could have altered the causative agent and antimicrobial sensitivity.^26^ Our series consisted of previously healthy infants brought spontaneously to a secondary level hospital by caretakers. We therefore consider this group to be representative of an etiological profile in an urban community of the city of São Paulo. Brazilians comprise a mixture of races, so that we were unable to establish if the prevalence of UTI is higher among Whites.^5^ We were also unable to define whether postectomy had any protecting effect against UTI in males, as previously reported.^5^ As this is not a common procedure in our community, it is likely that the prevalence of postectomy among males in our community is negligible.

In spite of the limitations of retrospective studies, our paper raises an alert about the need to investigate UTI with quantitative urine cultures in young infants that present with non-specific symptoms, and not to discard this diagnosis when a white blood cell count and/or CRP tests are within normal limits. Although *E. coli* was the main microbial agent, other Gram-negative microorganisms, such as *K. pneumoniae*, and Gram-positive cocci, such as *E. faecalis.* should be considered in the empirical approach to this condition. For additional knowledge about UTI in young infants, additional prospective studies are important to overcome the limiting factors described above, to assess urinary tract morphology in these cases, to evaluate the response to antimicrobial therapy and to observe further developments.
