# Erratum: Clinical and laboratory features of urinary tract infections in young infants

**DOI:** 10.1590/1678-4685-JBN-3602er

**Published:** 2019-02-25

In the article “Clinical and laboratory features of urinary tract infections in young infants”, with DOI code number http://dx.doi.org/10.1590/1678-4685-jbn-3602, published at Brazilian Journal of Nephrology, 40(1): 66-72, on the page 70:

Where it was written:

**DISCUSSION**

This study aims to bring clinical and laboratory knowledge about UTI in infants aged below 3 months in an urban community. Among this cohort, we found a high prevalence of UTI (12.5%) over the total number of urine cultures done in this group. There were three times more cases among females, concurring with previous studies.

Should read:

**DISCUSSION**

This study aims to bring clinical and laboratory knowledge about UTI in infants aged below 3 months in an urban community. Among this cohort, we found a high prevalence of UTI (12.5%) over the total number of urine cultures done in this group. There were three times more cases among males, concurring with previous studies.

